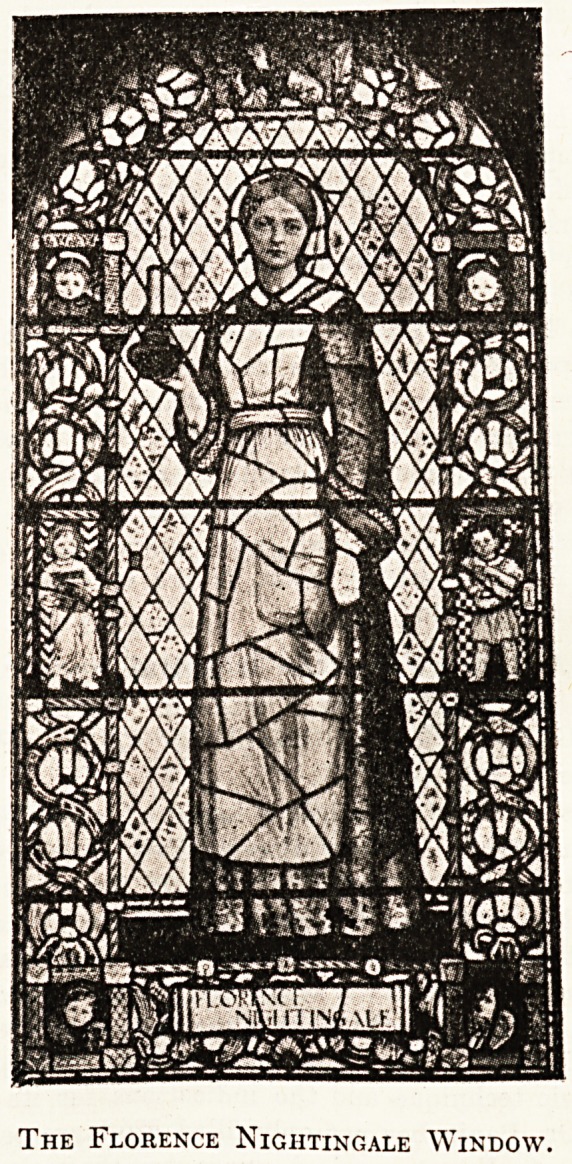# The Glasgow Royal Infirmary Chairman

**Published:** 1914-07-11

**Authors:** 


### The Glasgow Royal Infirmary Chairman.

The accompanying photograph of Mr. J. D. Hedderwick, LL.D., to whose fine record as chairman of the Glasgow Royal Infirmary we referred in The Hospital of June 27, page 343, cannot fail to be of interest. Mr.

Mr. J. D. Hedderwick, LL.D., Chairman of the Board of Managers, Royal Infirmary, Glasgow.

Hedderwick, unfortunately, has been on the sick list for over five months, suffering from severe neuralgia in the neck and head left from a bad attack of bronchitis. He will have to convalesce for some time to come, and it is to be hoped that his presence at the opening ceremony of the new Royal Infirmary on the 7th inst., which would have been incomplete without him, will have no bad effects on his health.

The Florence Nightingale Window.

We also publish a photograph of the "Florence Nightingale" window in the Glasgow Royal Infirmary chapel, which Mr. Hedderwick has presented. The work has been designed and executed by Mr. R. Anning Bell, A.R.A.

## Figures and Tables

**Figure f1:**
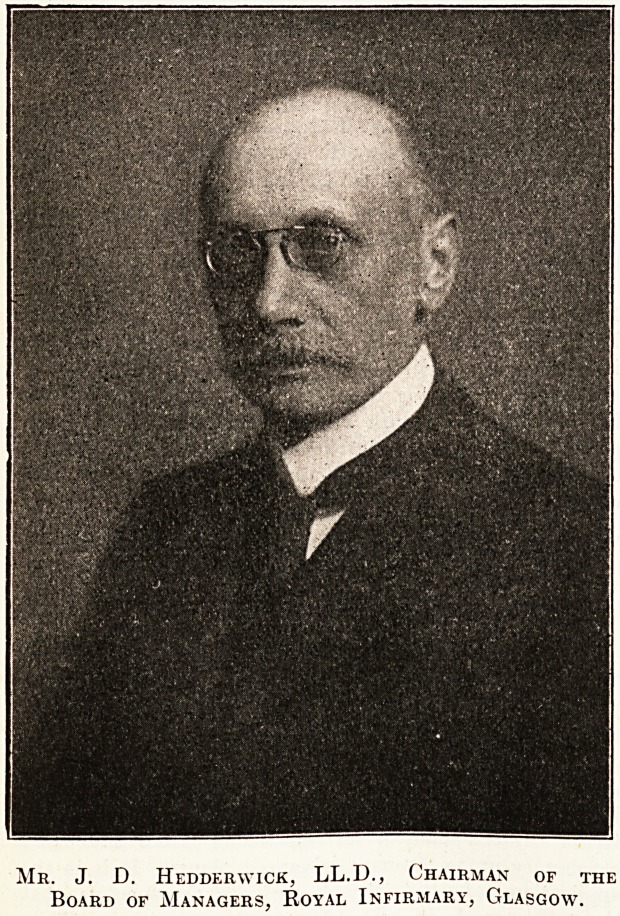


**Figure f2:**